# The Piraeus AIS dataset for large-scale maritime data analytics

**DOI:** 10.1016/j.dib.2021.107782

**Published:** 2022-01-03

**Authors:** Andreas Tritsarolis, Yannis Kontoulis, Yannis Theodoridis

**Affiliations:** Data Science Laboratory, Department of Informatics, University of Piraeus, Piraeus, Greece

**Keywords:** AIS data, Maritime, Piraeus, Big data analytics

## Abstract

The advent of Big Data and streaming technologies has resulted in a swarm of voluminous, heterogeneous information, especially in the domains of Internet of Things (IoT) and transportation. Focusing on the maritime field, in this paper, we present a dataset that contains vessel position information transmitted by vessels of different types and collected via the Automatic Identification System (AIS). The AIS dataset comes along with spatially and temporally correlated data about the vessels and the area of interest, including weather information. It covers a time span of over 2.5 years, from May 9th, 2017 to December 26th, 2019 and provides anonymised vessel positions within the wider area of the port of Piraeus (Greece), one of the busiest ports in Europe and worldwide. The dataset consists of over 244 million AIS records, an average of more than 10,000 records per hour, which makes it, to our knowledge, perhaps the largest and densest, to this extent of time, open AIS dataset to date, an ideal input for large-scale mobility data processing and analytics purposes.

## Specifications Table

**Table:** 

Subject	Data science
Specific subject area	Big Data Analytics
Type of data	Positions of vessels; Contextual; and Geographical data, related to maritime navigation
How data were acquired	The dataset was created by combining data collected by a terrestrial AIS receiver and publicly available datasets
Data format	Comma-Separated Values (CSV); ESRI Shapefile
Parameters for data collection	Spatial, temporal, and message-based filtering of received data
Description of data collection	AIS data collection and pre-processing, Guided search of open source maritime data on the web
Data source location	University of Piraeus, Piraeus, Greece. Lat/Lon: 37.9415, 23.6529
Data accessibility	The Piraeus AIS Dataset for Large-scale Maritime Data Analytics, URL: https://doi.org/10.5281/zenodo.5562629. Usage rights: Creative Commons Attribution-NonCommercial-ShareAlike 4.0 International (CC BY-NC-SA 4.0)

## Value of the Data

• The dataset is representative of operational information regarding a crowdy seaport, ideal for a variety of large-scale maritime data processing and analytics scenarios, including, among others, mobility profiling, offline, and streaming data analytics.
• The dataset can be useful to both practitioners and researchers working on projects regarding maritime data management and awareness.
• Due to its diversity and long spatio-temporal coverage, the dataset can support the training of vessel profiling models, from movement behaviour (e.g. routine vs. unusual movement) to activity profiles (e.g. anchoring vs. cruising vs. fishing, in case of fishery boats).

## 1 Data Description

In this section, we provide a description of the AIS dataset and its respective counterparts. Before we present the dataset, we provide necessary background information regarding vessel movement and AIS.

### 1.1 Background

Maritime mobility data consists of a variety of information, which can be very valuable in the context of the maritime domain. Towards this direction, the exploitation of positioning (tracking) messages is of great importance. The collected data can be used by a wide spectrum of maritime monitoring applications, providing a wealth of information useful for collision avoidance, traffic management, intelligent navigation, maritime situational awareness, as well as other real world applications. Among the various surveillance means used to identify and locate vessels at sea in real-time, the Automatic Identification System (AIS) has been made a mandatory standard on most common vessel types (cargo, passenger, fishing ships, etc.).

AIS is a surveillance technology that relies on the Global Positioning System (GPS) in conjunction with shipboard sensors (speedometer, compass, etc.). According to the International Maritime Organization’s (IMO) regulations,^1^ all international cargo vessels of at least 300 gross tonnage, along with all passenger vessels regardless of size are obliged to be equipped with operating AIS transmitters. Today, over half a million vessels use AIS to transmit their position.^2^

A vessel periodically broadcasts AIS messages that include kinematic information (position, speed, etc.), along with other static (type, flag, etc.) and voyage-related data (cargo, destination, etc.). Among the palette of information transmitted, of our main interest for the purpose of this paper is the vessel’s identifier, declared by its Maritime Mobile Service Identity (MMSI) number, its position recorded by GPS, i.e., polar coordinates in WGS-84 format, its course, heading, and speed.

Overall, there are 64 different types of AIS messages broadcast by AIS transceivers.^3^ In this paper, we focus on message types including location and (static) vessel information, which compose the vast majority of the AIS messages relayed; technically speaking, we consider AIS messages of ITU types 01, 02, 03, 05, 18, 19, and 24. In detail, types 01, 02, and 03 (class A position reports) provide a common reporting structure for navigational information, types 18 and 19 (class B position reports) deliver a brief report, complementary to types 01–03 for vessels using Class B transmitters, and types 05 and 24 (static data reports) refer to vessels’ static messages and are used in order to associate an MMSI with a name on either Class A or Class B equipment.

AIS signals are received by other AIS transponders (e.g., surrounding vessels equipped with AIS antenna, radio frequency towers, terrestrial and satellite AIS stations) within a typical range of 15–20 nautical miles. Especially for the cases where coverage through coastal AIS stations is not possible (e.g., at the oceans), satellite-based receivers are additionally used. As such, we distinguish between terrestrial AIS messages (T-AIS), continuously collected by onshore receiving stations, and satellite AIS messages (S-AIS), arriving in batches when satellites transfer data into a ground station.

Along with the position transmitted by the vessel, a timestamp *t* is also added by the AIS station that receives the signal. In normal operating conditions, the transmission rate varies between 2 and 10 s, depending on the vessel’s speed while underway, and is equal to 3 minutes when the ship is at anchor and stationary [1], [2]. On the other hand, static and voyage-related signals are transmitted with a lower frequency, i.e., approximately every 6 minutes.

For anonymisation purposes, the real identity of a vessel (MMSI) has been replaced by an artificial identifier and other privacy-related features (IMO number, name) have been removed. In other words, for the scope of this paper, an AIS kinematic signal is a record of format <t, vessel_id, lon, lat, heading, course, speed> and an AIS static signal is a record of format <vessel_id, country, type>.

### 1.2 Dataset coverage

**Fig. 1:**
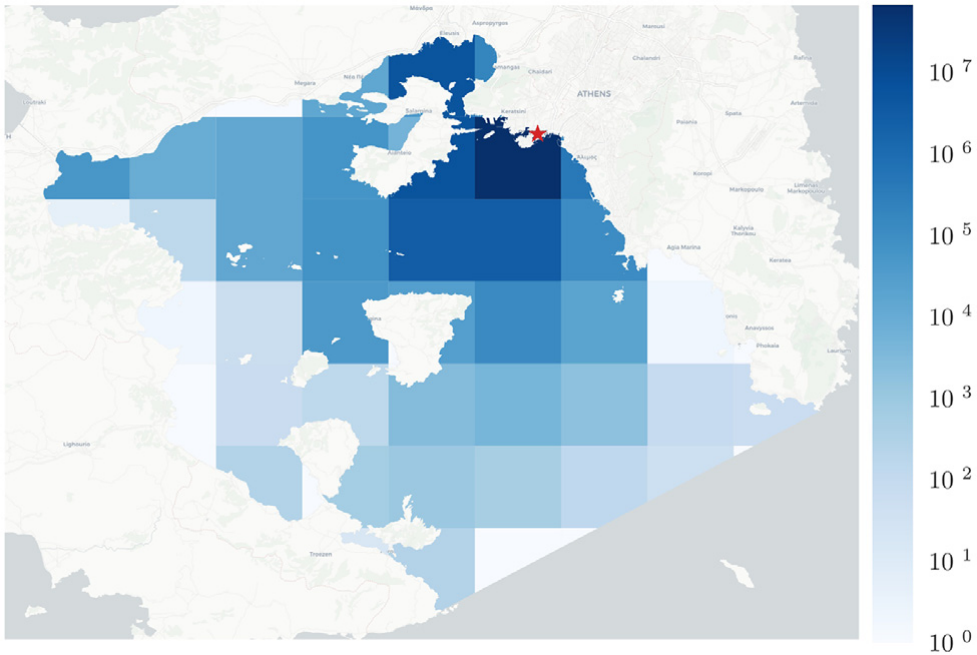
Location of AIS receiver (red star) and typical coverage (heatmap) within Saronic gulf.

**Fig. 2:**
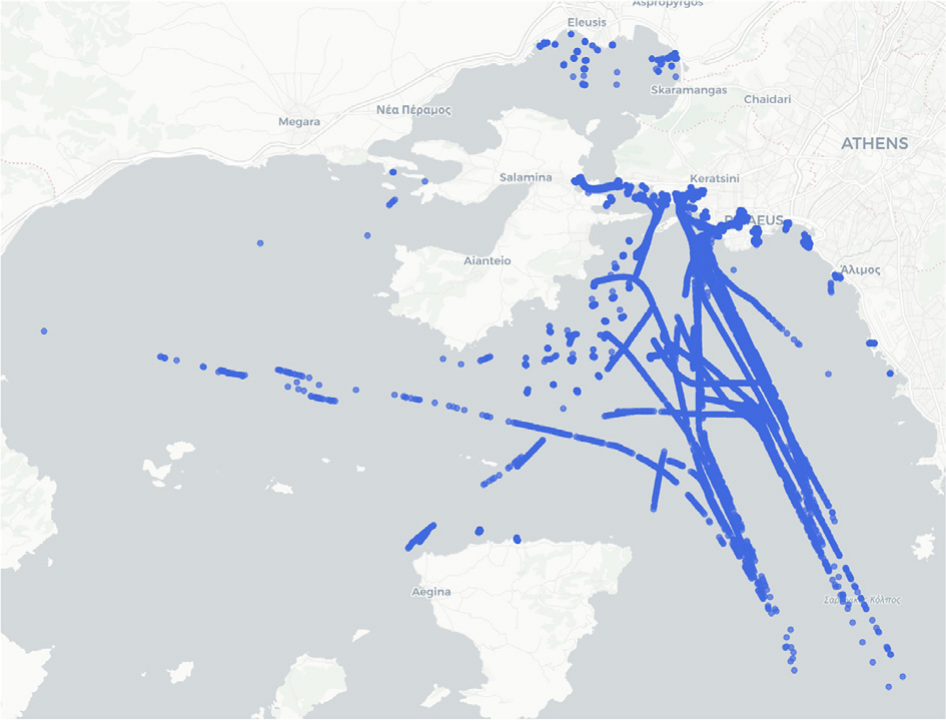
A Snapshot of the Piraeus Dataset: AIS positions captured on July 10th, 2018.

The AIS receiver used to gather the dataset is located at the roof of the central building of the University of Piraeus,^4^ in a line-of-sight distance of 1 km from the port of Piraeus. It consists of a commercial marine VHF antenna with frequency operating range of 156–162 MHz, interfaced to a Raspberry Pi server designed for commercial land-based monitoring of sea traffic. The receiver is linked to MarineTraffic^5^ maritime information service provider. Its spatial coverage corresponds to an area of approximately 10,000 sq.km., taking into consideration the recorded positions during the selected 32–month time period. Fig. 1 illustrates the location of the AIS receiver as well as its typical and maximum coverage (in terms of a heatmap that visualises the number of AIS kinematic signals detected), whereas Fig. 2 illustrates a snapshot of the dataset with the movement captured on an example date, July 10th, 2018, using the ST-Visions visualization tool [3].

## 2 Experimental Design, Materials and Methods

In the following sections, we describe the categories of data that constitute the dataset. Apart from navigation-related data (which is the core of the dataset), vessel-related (i.e., vessel registry), geo-related (islands, ports, coastline, etc.), and weather information as well as useful meta-data (in particular, annotated synopses) are also provided. Then, we explain the experimental process and conclude by presenting an abstract database model of the dataset.

### 2.1 AIS-related data

The AIS-related data are grouped in (i) kinematic and (ii) (static) vessel- related data. The AIS kinematic information consists of 32 files in CSV format; one file per month. Each row of these files contains a decoded AIS message, with the following information:

• timestamp: UNIX timestamp of the recieved AIS message, in ms.;
• vessel_id: vessel’s identifier (artificial, for anonymisation purposes);
• lon: longitude of vessel’s position, in angular units (WGS-84 format);
• lat: latitude of vessel’s position, in angular units (WGS-84 format);
• heading: vessel’s heading relative to true north, in degrees [0-360);
• course: vessel’s course over ground, in degrees [0-360); and
• speed: vessel’s speed, in knots.

**Fig. 3:**
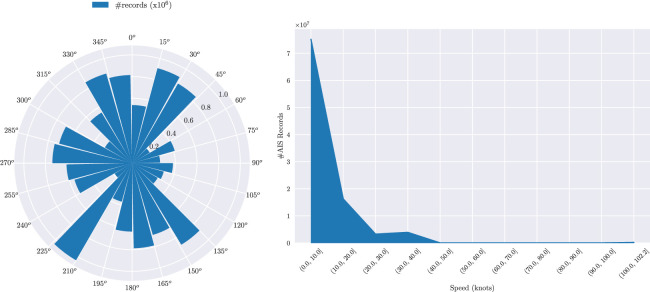
Statistics on navigation-related information: distribution of course over ground (left) and speed (right).

**Fig. 4:**
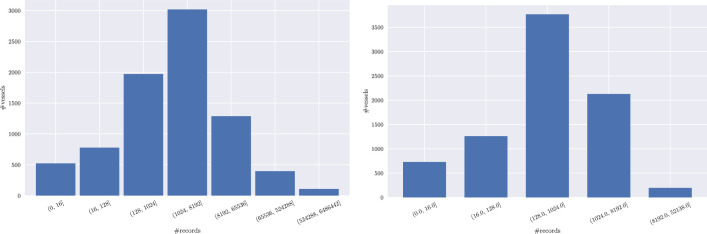
Statistics on navigation-related information (cont.): average number of records per vessel - i.e., unique (left) and per month (right).

Digging into the mobility information of this type of data, Fig. 3 illustrates two interesting charts about the recorded course over ground and speed, respectively, whereas Fig. 4 illustrates other two insightful statistics regarding the distribution of AIS messages per vessel (unique vessel_id) and per month, respectively.

On the other hand, the AIS static messages deliver information about vessels’ characteristics, which basically remain (relatively) unchanged over the duration of their voyage. In our dataset, we have included static information^6^ about country flag and vessel type, which is a 2-digit number representing a vessel’s general category and sub-category, respectively.^7^

Table 1 summarizes the AIS-related data in our dataset, consisting of three CSV files with kinematic, static, and vessel type information, respectively. In numbers, kinematic information exceeds 244 million records, thus making our dataset perhaps the largest, to our knowledge, open AIS source of information, compared e.g. with [4] (about 19 million AIS records in Brest, France) or [5] (14 million AIS records in the Baltic Sea).

**Table 1:** Summary of AIS-related data.

	Source	SRID	Coverage	Volume	Licence	Format and Description
AIS dynamic messages	Univ. Piraeus	EPSG:4326 (WGS84)	Dataset area	244,181,843 records (30 GB)	CC BY-NC-SA 4.0	32 CSV flat files containing AIS kinematic information
AIS static messages	Univ. Piraeus	—	Dataset area	6230 records (500 KB)	CC BY-NC-SA 4.0	CSV flat file containing vessels’ static information
Vessel types	MarineTraffic	—	—	100 records	CC BY-NC-SA 4.0	CSV flat file containing information about vessel types

### 2.2 Geo-related data

**Fig. 5:**
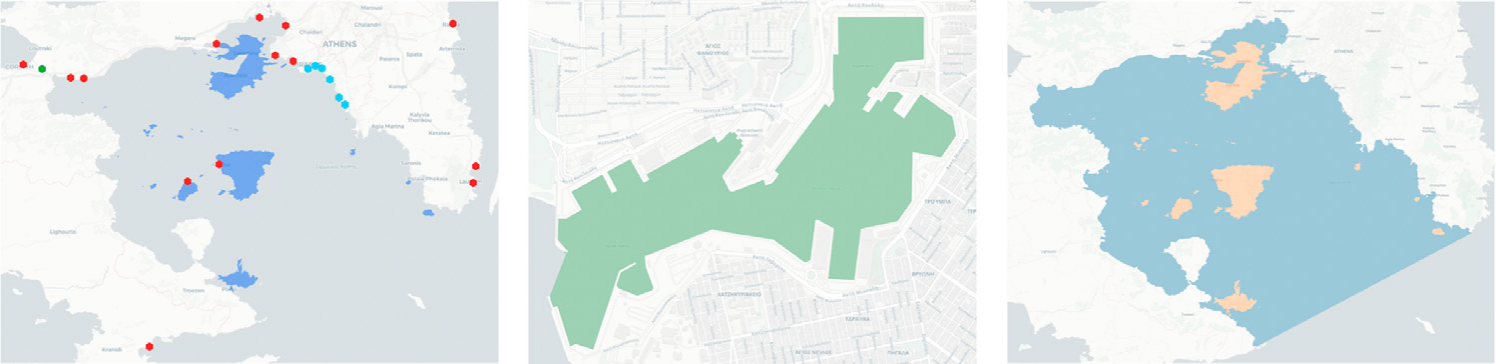
Saronic gulf ‘harbours’ (left); ‘PiraeusPort’ (center); and ‘islands’ - ‘territorialWaters’ (right) GIS layers.

We have enriched our dataset with seven GIS layers, organized in ESRI format, namely, *regions, harbours, port of Piraeus, islands, territorial waters, receiver location*, and *spatial coverage*. In the ‘regions’ layer, we have included the 13 regions of Greece, with Attica being the most notable since it covers Piraeus and the Saronic gulf islands nearby. The ‘harbours’ layer includes 14 ports, 6 marines, and 1 canal, located within the spatial coverage of the dataset; all are stored in point geometry, as illustrated by Fig. 5 (left). Especially for the port of Piraeus, which is of major importance for the usability of the dataset, we also provide its sketch in polygon format in a separate layer, as illustrated in Fig. 5 (center). The ‘islands’ and ‘territorialWaters’ layers include the polygons of 60 islands and a polygon mapping the territorial waters, respectively, in the area of interest, as Fig. 5 (right) illustrates. Regarding the last two layers, ‘receiver location’ and ‘spatial coverage’, they deal with the AIS station itself as their name declares.

Table 2 summarizes the geo-related data included in our dataset. As it appears in the table, some of the information comes from open sources, namely geodata.gov.gr^8^ and OpenStreetMap.^9^

**Table 2:** Summary of geographic-related data.

	Source	SRID	Coverage	Volume	Licence	Format and Description
Regions	geodata.gov.gr	EPSG:2100	Greece	13 polygons (5.3 MB)	CC BY 3.0	ESRI shapefile containing the regions of Greece
Harbours	(own processing)	EPSG:4326 (WGS84)	Dataset area	21 points (28 kB)	CC BY-NC-SA 4.0	ESRI shapefile containing the major ports of Saronic Gulf
Piraeus Port	OpenStreetMap	EPSG:4326 (WGS84)	Dataset Area	1 polygon (24 kB)	ODbL 1.0	ESRI Shapefile containing the area of the Port of Piraeus
Islands	OpenStreetMap	EPSG:4326 (WGS84)	Dataset Area	60 polygons (276 kB)	ODbL 1.0	ESRI shapefile containing the islands of Saronic Gulf
Territorial Waters	OpenStreetMap	EPSG:4326 (WGS84)	Dataset Area	1 polygon (605.8 kB)	ODbL 1.0	ESRI Shapefile containing the area of Saronic Gulf
Receiver location	(own processing)	EPSG:4326 (WGS84)	Dataset area	1 point (469 bytes)	CC BY-NC-SA 4.0	ESRI shapefile containing the position of AIS receiver
Spatial Coverage	(own processing)	EPSG:4326 (WGS84)	Piraeus and Saronic Gulf	76 polygons (620.2 kB)	CC BY-NC-SA 4.0	ESRI shapefile containing the spatial coverage of the dataset (in grid cells)

### 2.3 Weather data

Linking position data with weather-related information can be extremely useful for data analytics purposes, e.g., by providing insights regarding vessels’ movement and assisting in explaining abnormal situations. For instance, sea and weather conditions can force vessels to change their direction or adjust their usual routine. In addition, weather information can also be used to characterize seasonal trends in traffic routes and to contextualise vessels’ kinematics, such as speed.

**Fig. 6:**
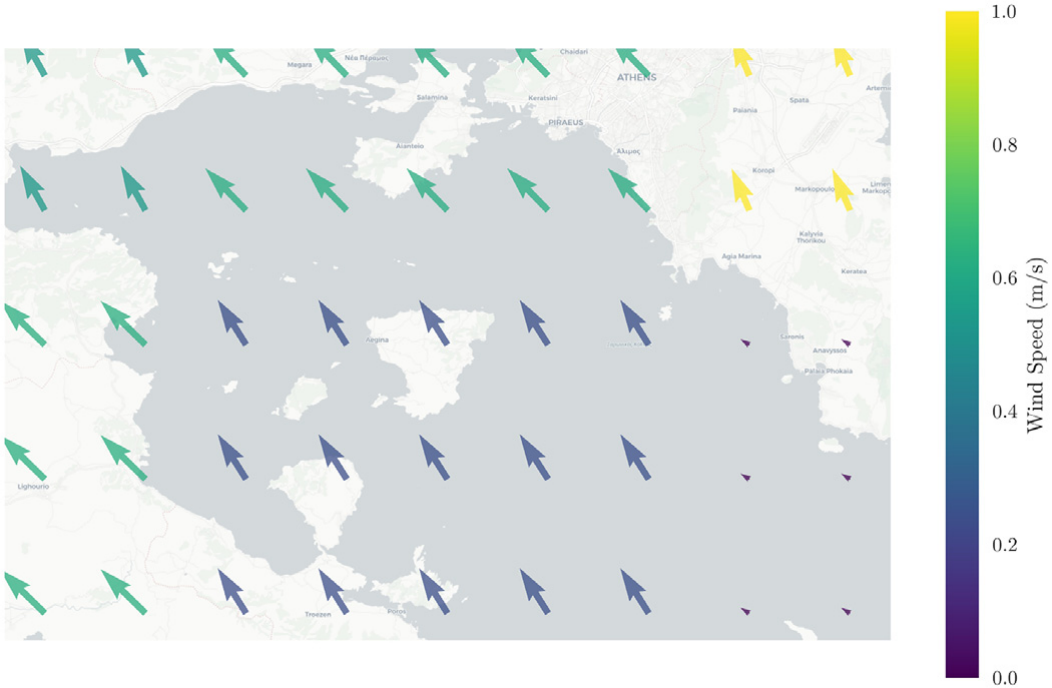
Plot of wind direction and speed, July 23, 2018, 12:00.

In our dataset, we have included weather observations recorded by National Oceanic and Atmospheric Administration (NOAA),^10^ organized in GRIB files.^11^ GRIB files with forecast weather data are publicly offered by NOAA and are freely available for download. For the purposes of our dataset, we used Weather Integrator,^12^ an open-source tool developed in our Lab for integrating mobility data with weather information  [6]. In particular, we partitioned the spatial area of interest in a 9x5 spatial grid and the temporal interval of interest in 6 h periods and, for each such record, we gathered 14 weather features related to temperature, air pressure, humidity, wind gust speed, visibility, precipitation, wind direction, and wind speed. For instance, Fig. 6 illustrates wind direction and speed during a certain time period (July 2, 2018, 06:00–12:00); wind speed is visualised by the respective color and wind direction is represented by the arrow heading.

The above information is organized in 32 ESRI shapefiles, one per month. Each shapefile contains 19 columns: 14 columns for the weather features plus 2 columns for the geometry (point) of the centroid of the spatial cell plus 2 columns for the timestamp (in UNIX and datetime formats) plus 1 column for the record id. Table 3 summarizes the weather data included in our dataset.

**Table 3:** Summary of weather-related data.

	Source	SRID	Coverage	Volume	Licence	Format and Description
Weather	NOAA	EPSG:4326 (WGS84)	Dataset area	194,400 records (222 MB)	NOAA Data Policy	32 ESRI Shapefiles containing spatial- temporal intervals along with their respective weather features

### 2.4 Trajectory synopses

Given the fact that vessels are expected to follow (in most cases) predictable routes in the sea, a sufficient number of positions along trajectory segments could be ignored, retaining only those positions conveying salient mobility events. The identification of these *critical points* [7] is based on the observation of changes on the vessel’s motion pattern (whether it is in motion or stationary, whether it changes heading or speed, etc.). Through this information compression process, it becomes possible to reconstruct the traces of the vessels by only using notable trajectory summaries, while maintaining high level of quality with regard to the resulting trajectory approximation.

**Fig. 7:**
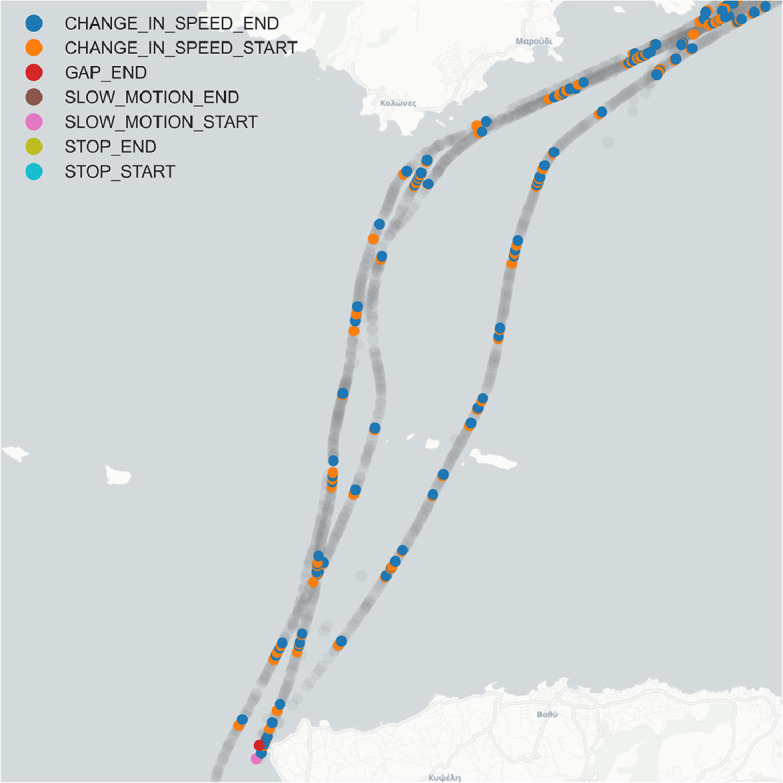
Vessel trajectory (gray) and its corresponding critial points – synopses referring to a vessel’s voyage on July 23, 2018.

For the purposes of enriching our dataset with useful synopses, we have used Synopses Generator,^13^ another open source tool developed in our Lab for summarizing sequences of sampled positions of moving objects, and output summaries, i.e., a much smaller number of positions, which are associated with annotations from the following set: {STOP, CHANGE_IN_SPEED, SLOW_MOTION, GAP, CHANGE_IN_HEADING, NOISE}. Indicatively, we provide 20 CSV files, one for each available month (May 2017 - Dec. 2018), containing the produced synopses. Table 4 summarizes the trajectory synopses data included in our dataset, while Fig. 7 illustrates the full trajectory of a vessel at a certain point in time compared to its respective synopses.

**Table 4:** Summary of synopses meta-data.

	Source	SRID	Coverage	Volume	Licence	Format and Description
Trajectory Synopses	(own processing)	EPSG:4326 (WGS84)	Dataset area	9,452,358 records (2.2 GB)	CC BY-NC-SA 4.0	20 CSV flat files containing vessels’ trajectory synopses

### 2.5 Experimental process

The process we followed in order to build our dataset is outlined in the following steps: (i) AIS message decoding, (ii) AIS position cleansing and storage, (iii) enrichment of vessel positions with weather information, and (iv) synopses generation out of vessels’ AIS positions. In particular^14^:

*AIS message decoding*: There are plenty of software libraries and tools available to decode and parse AIS messages. We used AisLib,^15^ a Java-based message decoder, compliant with ITU 1371 (NMEA armoured AIS messages). Decoding is an online precess and takes 20 ms, on the average, per message.

*AIS position cleansing and storage*: It is not rare that AIS messages contain noise, for instance, due to erroneous GPS input. In general, cleansing is a typical pre-processing step when handling mobility data [8]. In our (simple) cleansing process, as soon as we receive a position, we keep it only if it falls inside the area of interest (cf. Fig. 1) and, then, we store it in the respective CSV file. Cleansing and storage take 22 ms, on the average, per message.

*Enrichment with weather information*: As already discussed, we used an open source tool developed in our group (Weather Integrator). In particular, we streamed the (cleansed) AIS positions, combined them with archived GRIB files (already downloaded from NoAA), and associated each position with the MBB that covers it. For each position, this operation takes 16 ms, on average.

*Synopses generation*: As already discussed, also for this step we used an open-source tool developed in our group (Synopses Generator). In particular, we streamed the (cleansed) AIS positions, processed this stream using this tool, and produced synopses. This step takes 100 ms, on average, per message.

### 2.6 All-in-one: DB abstract model

**Fig. 8:**
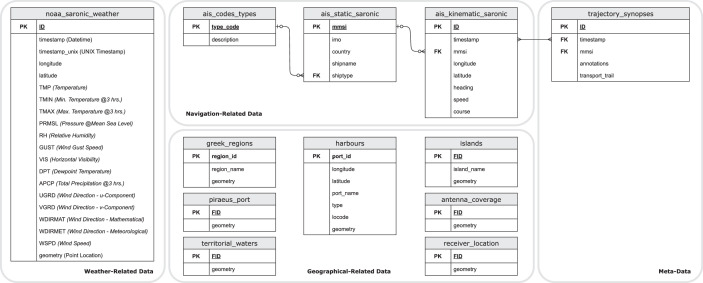
Abstract DB model of the Piraeus AIS dataset.

Closing the presentation of our dataset, we provide the respective spatial database model, illustrated in Fig. 8. Each bounding rectangle corresponds to a type of information, as described in the sections above.

Summarizing, in this paper we presented a large open AIS dataset consisting of over 244 million positions of vessels sailing in the wider area of port of Piraeus, Greece, during a 32-months period. The position data were enriched by (static) vessel-related information as well as surrounding information about the geography and the weather conditions and synopses over the vessel trajectory. In total, the dataset is composed by 46 flat data files (in CSV format) and 39 geographic layers (in ESRI shapefile format), with an overall size of approximately 20 GB.

We hope that the dataset will turn out to be a valuable resource for researchers and practitioners in the maritime field. Although it is self-standing, it can also be used in relation with other related open sources, e.g., [9]. We also plan to expand our dataset in periodical batches as long as our AIS station remains in operation.

## Ethics Statement

The authors have all permissions and consent for sharing this AIS dataset. The AIS dataset has been fully anonymised, fully complying with the data redistribution platform (zenodo.com) it is available at.

## CRediT authorship contribution statement

**Andreas Tritsarolis:** Data curation, Visualization, Software, Writing – original draft. **Yannis Kontoulis:** Data curation, Investigation, Writing – original draft. **Yannis Theodoridis:** Supervision, Writing – review & editing.

## Declaration of Competing Interest

The authors have no competing interests to declare that are relevant to the content of this article.

## Appendix Supplementary materials

Image, application 1

## Footnotes

1 Guidance on the use of AIS Application-Specific Messages, URL: http://vislab-ccom.unh.edu/~schwehr/papers/2010-IMO-SN.1-Circ.289.pdf.

2 source: https://www.marinetraffic.com/blog/seven-things-know-ais/

3 International Telecommunication Union (ITU), Technical characteristics for an Automatic Identification Systemusing time division multiple access in the VHF maritime mobile frequency band (Recommendation M.1371-5), February2014, p. 148, URL: (https://www.itu.int/rec/R-REC-M.1371-5-201402-I)

4 A real-time visualization of the vessels tracked by the University of Piraeus’ receiver is available here: https://www.datastories.org/unipi-ais/.

5 URL: https://www.marinetraffic.com/en/ais/details/stations/2782

6 Due to the fact that this information is usually entered manually rather that automatically collected by sensors, it is subject to errors, therefore, it should be thoroughly cross-checked before any analytics process relying on it is performed.

7 Some popular types include 30 (Fishing), 36 (Sailing vessel), 37 (Pleasure craft), 60–69 (Passenger), 70–79 (Cargo), and 80–89 (Tanker). Source: https://help.marinetraffic.com/hc/en-us/articles/205579997-What-is-the-significance-of-the-AIS-Shiptype-number.

8 http://geodata.gov.gr/en/dataset/28121643-d977-48eb-a8ca-a6fac6b4af6d/resource/7c80a2c1-93b7-4814-9fc4-245e775acaa6

9 https://nominatim.openstreetmap.org/ui/details.html?osmtype=R&osmid=9135811.

10 https://www.noaa.gov/

11 GRIB is a standardized binary data format by the World Meteorological Organization’s Commission for Basic Systems, commonly used in meteorology to store historical and forecast weather data.

12 URL: https://github.com/DataStories-UniPi/Trajectory-Synopses-Generator.

13 URL: https://github.com/DataStories-UniPi/Trajectory-Synopses-Generator.

14 The process presented below operated on a server running Debian Linux “Wheezy” 7.5 amd64 with 48 GB of RAM and two Intel Xeon X5675 processors at 3.07GHz

15 URL: https://github.com/dma-ais/AisLib.
