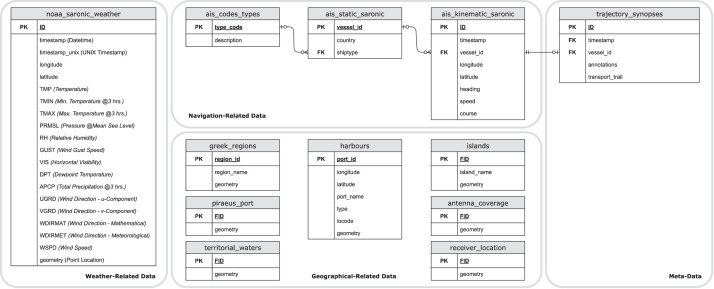# Corrigendum to `The Piraeus AIS dataset for large-scale maritime data analytics' [Data in Brief, 40 (2022), 107,782/DIB_107782]

**DOI:** 10.1016/j.dib.2022.107940

**Published:** 2022-02-10

**Authors:** A. Tritsarolis, Y. Kontoulis, Y. Theodoridis

**Affiliations:** Data Science Laboratory, Department of Informatics, University of Piraeus, Piraeus, Greece

The authors regret to declare that a figure that appears in the published article (in particular, Fig. 8) is not the correct one and may result to misunderstanding (especially when compared with the dataset that accompanies the article). The correct version of Fig. 8 is attached in this corrigendum. The authors would like to apologise for any inconvenience caused.

**Figure:**